# Ecologically relevant choanoflagellates collected from hypoxic water masses of the Baltic Sea have untypical mitochondrial cristae

**DOI:** 10.1186/1471-2180-12-271

**Published:** 2012-11-21

**Authors:** Claudia Wylezich, Sergey A Karpov, Alexander P Mylnikov, Ruth Anderson, Klaus Jürgens

**Affiliations:** 1 IOW-Leibniz Institute for Baltic Sea Research Warnemünde, Rostock, Germany; 2 Zoological Institute RAS and St. Petersburg State University, St. Petersburg, Russia; 3 Institute for the Biology of Inland Waters, Russian Academy of Sciences, Borok, Russia

## Abstract

**Background:**

Protist communities inhabiting oxygen depleted waters have so far been characterized through both microscopical observations and sequence based techniques. However, the lack of cultures for abundant taxa severely hampers our knowledge on the morphology, ecology and energy metabolism of hypoxic protists. Cultivation of such protists has been unsuccessful in most cases, and has never yet succeeded for choanoflagellates, even though these small bacterivorous flagellates are known to be ecologically relevant components of aquatic protist communities.

**Results:**

Quantitative data for choanoflagellates and the vertical distribution of *Codosiga* spp. at Gotland and Landsort Deep (Baltic Sea) indicate its preference for oxygen-depleted zones. Strains isolated and cultivated from these habitats revealed ultrastructural peculiarities such as mitochondria showing tubular cristae never seen before for choanoflagellates, and the first observation of intracellular prokaryotes in choanoflagellates*.* Analysis of their partial 28S rRNA gene sequence complements the description of two new species, *Codosiga minima* n. sp. and *C. balthica* n. sp. These are closely related with but well separated from *C. gracilis* (*C. balthica* and *C. minima p*-distance to *C. gracilis* 4.8% and 11.6%, respectively). In phylogenetic analyses the 18S rRNA gene sequences branch off together with environmental sequences from hypoxic habitats resulting in a wide cluster of hypoxic *Codosiga* relatives so far only known from environmental sequencing approaches.

**Conclusions:**

Here, we establish the morphological and ultrastructural identity of an environmental choanoflagellate lineage. Data from microscopical observations, supplemented by findings from previous culture-independent methods, indicate that *C. balthica* is likely an ecologically relevant player of Baltic Sea hypoxic waters. The possession of derived mitochondria could be an adaptation to life in hypoxic environments periodically influenced by small-scale mixing events and changing oxygen content allowing the reduction of oxygen consuming components. In view of the intricacy of isolating and cultivating choanoflagellates, the two new cultured species represent an important advance to the understanding of the ecology of this group, and mechanisms of adaptations to hypoxia in protists in general.

## Background

Choanoflagellates are colourless, free-living, exclusively heterotrophic protists that are characterized by a single anterior flagellum surrounded by a collar of microvilli; and flat cristae in the mitochondria [1]. These unikont flagellates form the sister taxon of metazoans as seen by sequence analyses [2-4]. Within the choanoflagellates, three families were originally distinguished based on morphology: Acanthoecidae Norris, 1965; Salpingoecidae Kent, 1880; and Codonosigidae Kent, 1880 (synonym Monosigidae Zhukov et Karpov, 1985). Recent taxonomic revision based on multigene analysis states that the class Choanoflagellatea Kent, 1880 comprises two orders: 1) Craspedida, with a single family Salpingoecidae (including the aloricate choanoflagellates of the former Codonosigidae and Salpingoecidae families); and 2) Acanthoecida, with the families Acanthoecidae and Stephanoecidae [5,6]. Choanoflagellates normally constitute 5 to 40% of the average heterotrophic nanoflagellates (HNF) biomass in oxygenated pelagic habitats [7,8]. They have also been detected in hypoxic (oxygen-deficient) water masses [9] and can constitute a significant proportion of total HNF biomass, reaching for example 10–40% in hypoxic water masses of the Baltic Sea [10]. Especially in Gotland Deep, the biomass of exclusively aloricate choanoflagellates can clearly exceed 40% [10]. However, to date, few choanoflagellate species have been successfully cultured [5], and none for hypoxic environments, limiting knowledge on the ecology of this ecologically relevant protist group.

Clone library based approaches have produced many novel sequence types during the last decade, enhancing our knowledge of protist species richness and diversity [11,12]. However, morphological and quantitative data of microscopical life observations and cell counts are often hard to match with such environmental sequences. In some recent cases it has been possible to assign new described species to novel protistan lineages only known from culture-independent sequence investigations [13-15]. Many environmental sequences (18S rRNA) in public databases cluster within the choanoflagellates. A recent re-analysis of published environmental sequences belonging to this group [16,17] provided evidence for only a low correspondence between these sequences and sequences obtained from cultures. Clonal sequences from hypoxic environments (here referring to suboxic to anoxic/sulfidic conditions) have also been found to often cluster within the choanoflagellates. For instance, sequences from the anoxic Framvaren Fjord [18] branch off near *Diaphanoeca grandis* (Stephanoecidae); and clonal sequences found in the hypersaline Mediterranean L’Atalante Basin constitute the novel cluster F within the Acanthoecidae [16,19]. Stock *et al*. [20] also detected novel sequences in the redoxcline of the periodically anoxic Gotland Deep (central Baltic Sea), which branched within the Craspedida cluster A [16]. However, only a small fraction of choanoflagellates known at a sequence level have been isolated and maintained in culture to date, and none so far was derived from hypoxic marine environments. Thus, the morphology, ultrastructure and physiological strategies of these choanoflagellates from hypoxic environments remain unexplored.

The Baltic Sea is one of the largest brackish water basins in the world. A stable halocline separates the water column into an upper oxygenated layer and underlying oxygen deficient and anoxic/sulfidic layers in the deeper basins (e.g., Gotland and Landsort Deep). Protist communities inhabiting these oxygen depleted layers have been characterized so far by microscopical counting of stained specimens [21-23] and clone library investigations [20]. However, in contrast to well characterized prokaryotic communities inhabiting these zones [24-26], little is known on the ecology and ultrastructure of individual protist groups living there.

The aim of this survey was to successfully isolate and cultivate ecologically relevant protist strains from hypoxic water masses of the Baltic Sea and characterize the morphological and ultrastructural traits that could allow them to succeed in these environments. In the present study we present two successfully cultured choanoflagellate isolates of the genus *Codosiga*, which present mitochondria with tubular cristae and endobiotic bacteria, never seen before for choanoflagellates, which could represent an adaptation to life in an environment with fluctuating oxygen content.

## Results

### Vertical distribution and abundance of choanoflagellates

**Figure 1:**
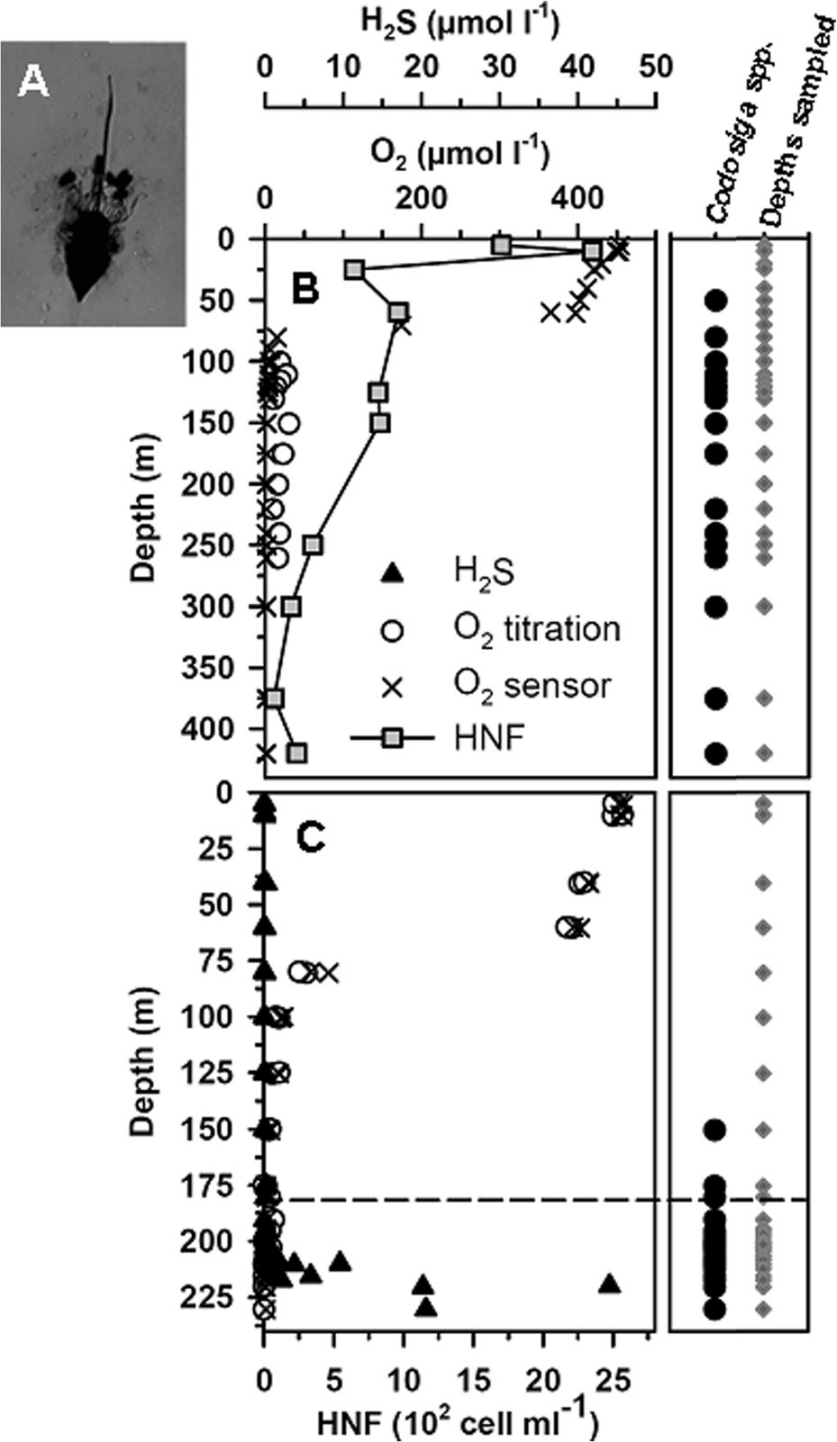
**Vertical distribution of *Codosiga *spp. indentified in May 2005, and assessment of their presence (black circles) / absence (no symbol) at different depths (grey diamonds) throughout the whole water column of Landsort Deep (B) and Gotland Deep (C).** Oxygen concentrations (measured by titration and by the oxygen sensor on the CTD) and hydrogen sulfide concentrations (only available for Gotland Deep) are also shown, along with cell-counts for Landsort Deep. Data were pooled for several different CTD casts. The dashed line represents the chemocline. *Codosiga* spp. was identified by life observations and scanning electron microscopy as shown **(A).**

**Figure 2:**
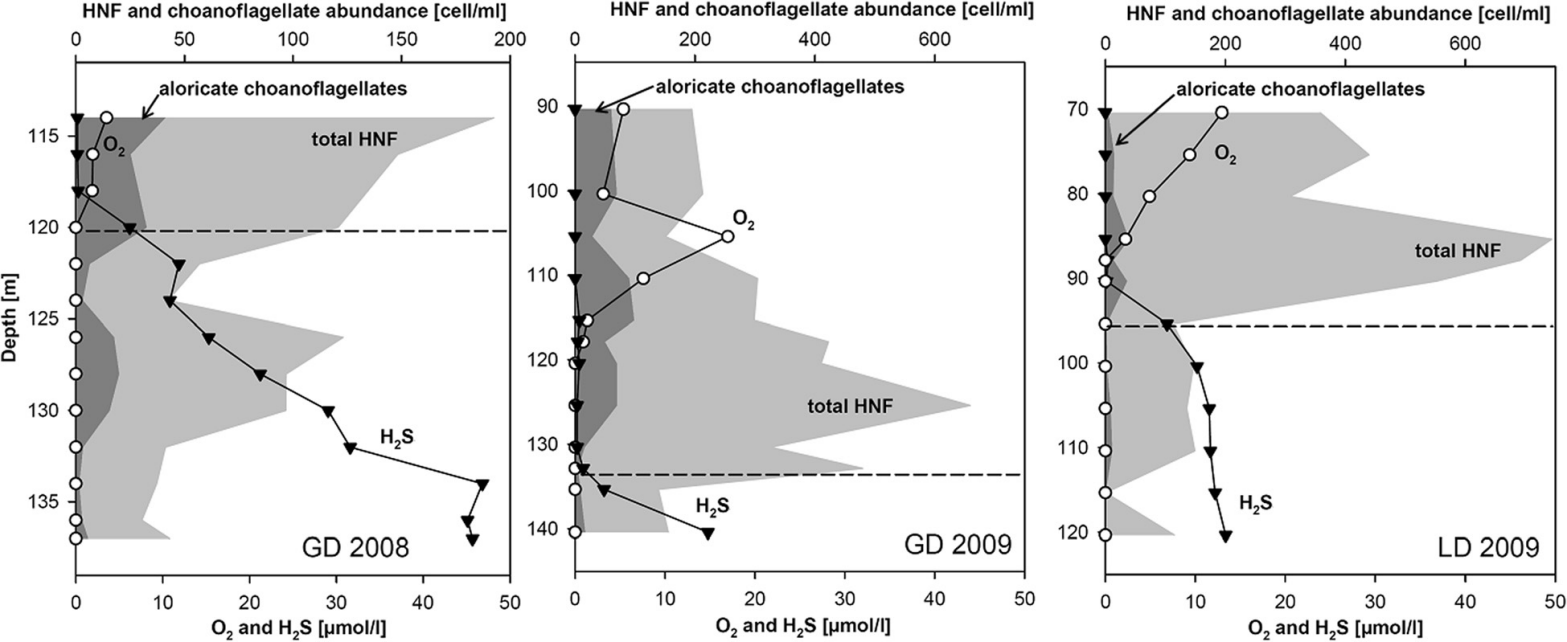
**Abundance of heterotrophic nanoflagellates (light grey) and relative abundance of naked choanoflagellates (dark grey) in redoxclines of Gotland Deep in 2008 (GD 2008) and 2009 (GD 2009) and Landsort Deep 2009 (LD 2009) based on epifluorescence microscopy.** The horizontal dashed line represents the first appearance of hydrogen sulfide (chemocline). Note the changes in the scale of some axis between the two years.

In 2005, an analysis of *Codosiga* spp. and its vertical distribution was conducted through light and electron microscopy (Figure 1A) for the whole water column of Landsort and Gotland Deep (Figure 1B, C). The detected *Codosiga* specimens showed a preference for suboxic and anoxic water layers in both sites. In Gotland Deep the cells were mainly detected in sulfidic waters below the chemocline (defined by the first appearance of hydrogen sulfide). The HNF cell counts from the redoxclines in 2008 and 2009 (Figure 2) are shown as the abundance of total heterotrophic flagellates and the relative proportion of aloricate choanoflagellates (including *Codosiga* and other naked genera). Choanoflagellates were numerically important components in Gotland Deep, but represented only a small fraction of total HNF in Landsort Deep (Figure 2). Their abundance was highest at suboxic and interface depths ranging from 20 to 30% of total HNF counts in Gotland Deep and about 5% Landsort Deep.

### Phylogenetic reconstructions using ribosomal gene sequences

Nearly complete 18S rRNA gene sequences were obtained for both strain IOW73 (1748 base pairs in length), and strain IOW94 (1783 base pairs). Additionally, we generated partial 28S rRNA sequences for both strains to enable comparison with *Codosiga gracilis* from GenBank (the 18S rRNA sequence is missing for this unique *Codosiga* culture, see [6]). The 28S sequences obtained, including the divergent D1-D6 regions, possessed a length of 1620 and 1612 base pairs for strain IOW73 and strain IOW94, respectively.

**Figure 3:**
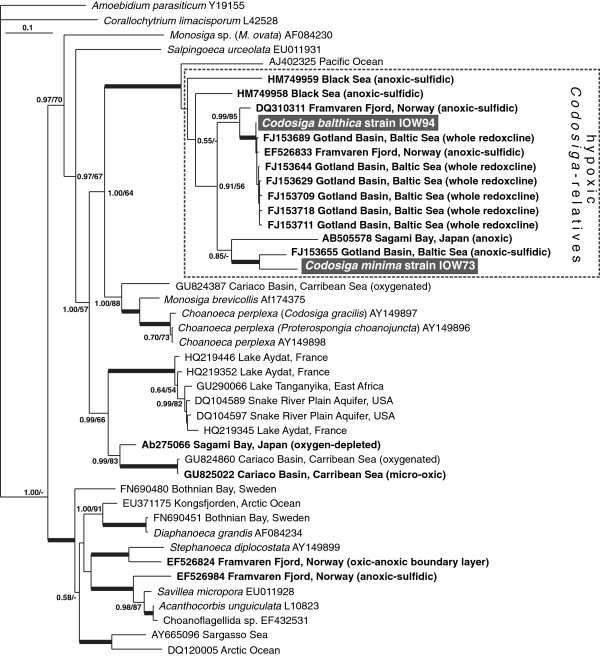
**Phylogenetic relationships of choanoflagellate strains isolated within this study to environmental sequences from hypoxic habitats based on partial 18S rRNA sequences using MrBayes.** New species are presented in white bold characters; environmental clonal sequences of hypoxic habitats are shown in bold face letters. Posterior probability and bootstrap values above 0.5 and 50 are indicated. Values above 0.99 and 99 are presented as bold face branches. Scale bar represents 0.1 mutations per position. *Amoebidium parasiticum* (Ichthyosporea) was used as outgroup representative.

Strains IOW73 and IOW94 belong to the Salpingoecidae according to [6] and branched off with clade 1 by Carr *et al*. [5], and clade A by del Campo & Massana [16]. The 18S rRNA tree (Figure 3) additionally contains environmental sequences from different habitats closely related to clade A. The *Codosiga* sequences form a well supported clade with sequences from hypoxic habitats such as the Baltic Sea (Gotland Deep), Framvaren Fjord, the Black Sea and Sagami Bay, Japan. The only exceptional sequence in this clade, that was not isolated from hypoxic environment, is AJ402325 from the Pacific [27] which forms the basal branch. We were able to establish cultures for two further strains, IOW74 (Gotland Deep, 208 m) and IOW75 (Landsort Deep, 260 m), whose short 18S rRNA sequence fragments are identical to strain IOW73 (data not shown).

**Figure 4:**
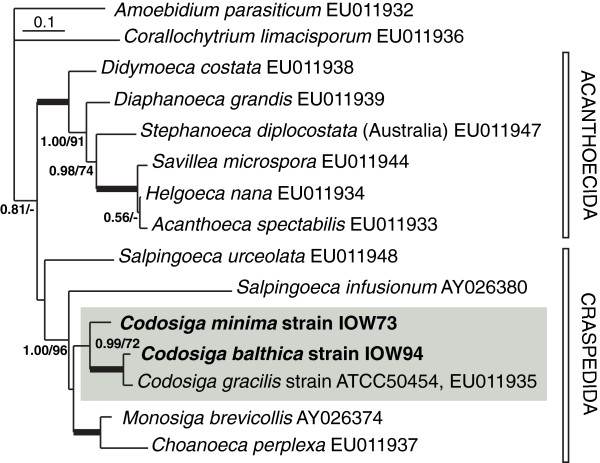
**Phylogenetic relationship of *Codosiga balthica *and *C. minima *within Craspedida based on partial 28S rRNA sequences excluding the fast evolving divergent D2 region using MrBayes.** Posterior probability and bootstrap values above 0.5 and 50 are shown. Scale bar represents 0.1 mutations per position. Values above 0.99 and 99 are presented as bold face branches. Scale bar represents 0.1 mutations per position. *Amoebidium parasiticum* (Ichthyosporea) was used as outgroup representative.

The phylogenetic tree based on partial 28S rRNA gene sequences, excluding the highly divergent D2 region, shows a well established branching order in the Craspedida and Acanthoecida (Figure 4). Sequences of our new isolates are closely related to *Codosiga gracilis* ATCC50454, rendering the genus *Codosiga* monophyletic. Strain IOW94 is more closely related to *C. gracilis* (*p*-distance 4.8%) than IOW73 (*p*-distance to *C. gracilis* 11.6%).

### Cultivation and morphology

**Figure 5:**
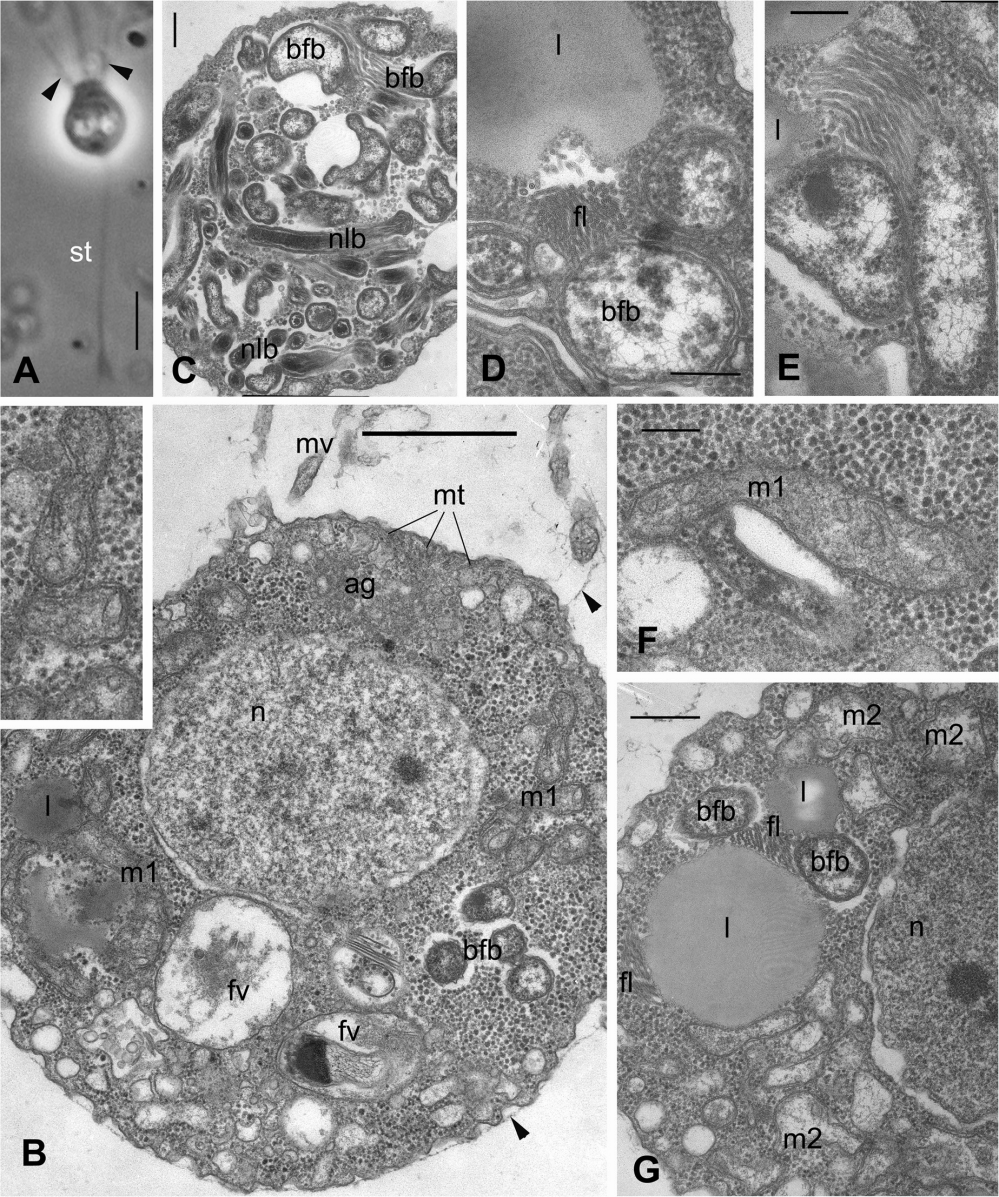
***Codosiga balthica *n. sp. strain IOW94.** Light **(A)** and transmission electron **(B-G)** micrographs. **A.** Single cell on the stalk (st), living material under phase contrast. Arrowheads show the whiskers. **B.** Longitudinal section through the cell covered with delicate sheath (arrowheads); insert: enlarged mitochondria of class 1 (m1) with tubular/saccular cristae. **C.** Cytoplasm at cell posterior filled with endobiotic bacteria. **D–E.** structure of large flagellated bacteria with flagellar at cross section **(D)** and longitudinal section **(E)**. **F.** mitochondria class 1 (m1) with tubular/saccular cristae. **G.** mitochondria class 2 (m2) structure with tubular cristae and lipid globule association with bfb. Scale bars: A – 3 μm, B – 1 μm, C-F – 200 nm, G – 400 nm.

**Figure 6:**
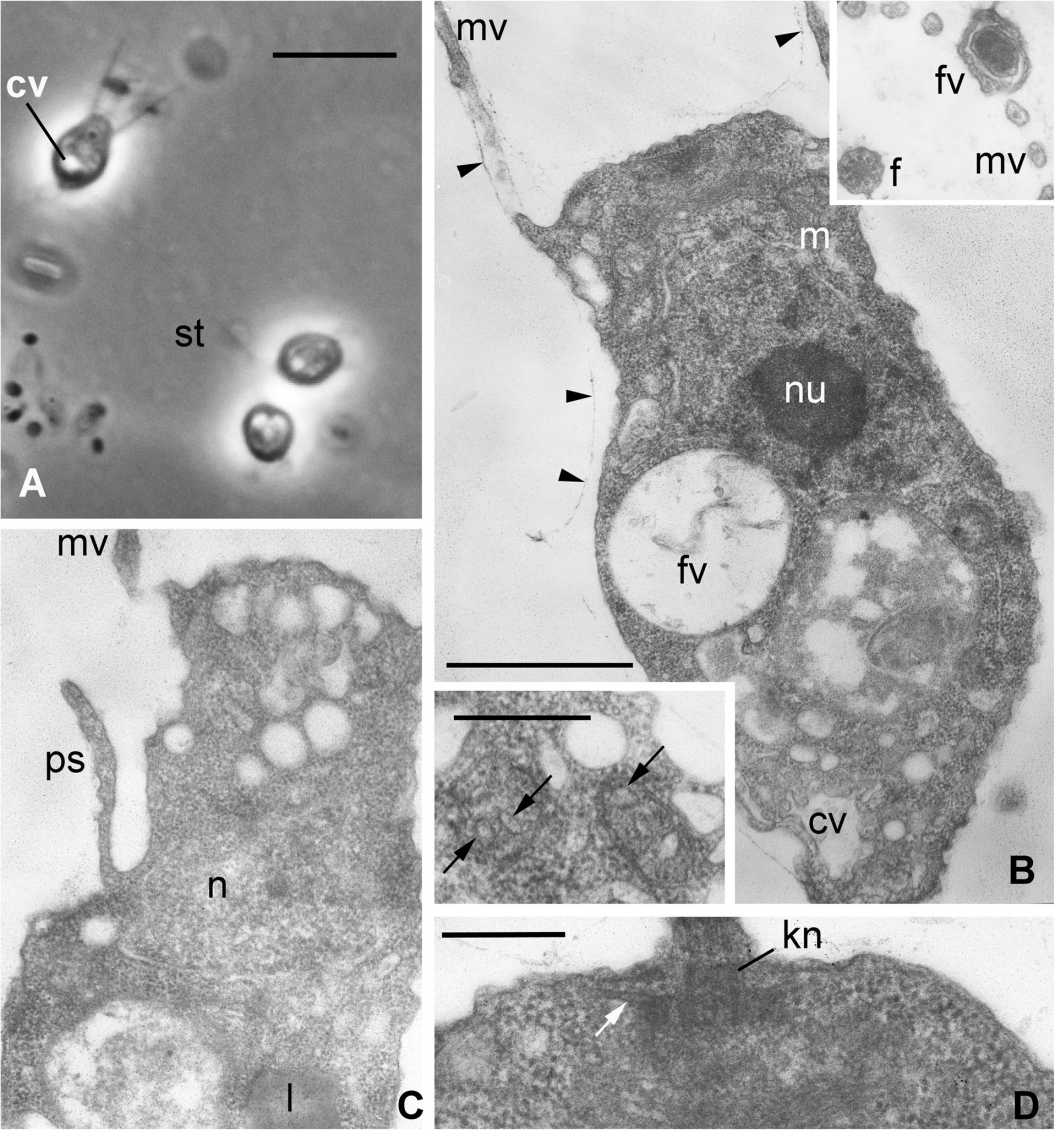
***Codosiga minima *n. sp. strain IOW73.** Light **(A)** and transmission electron **(B-G)** micrographs. **A.** Single cell and two-cell colony with a stalk (st), living material under phase contrast. **B.** Longitudinal section of the cell, arrowheads show a delicate sheath around the cell body and proximal part of collar microvilli (mv). Insert upper right: transversal section through the collar with food vacuole (fv) with bacterium at outer side of the collar. Insert down left: two mitochondrial profiles with tube-like cristae (arrows). **C.** Longitudinal section of feeding cell in the colony: pseudopodium (ps) arises from the neck. **D.** Longitudinal section of flagellar kinetosome (kn) with one row of radiating microtubules (arrows). Scale bars in **A** = 4 μm, **B** (+ upper insert), **C** = 2 μm, B (down insert), **D** = 500 nm.

Choanoflagellate cultures were maintained under oxic conditions. The culture development in both strains was similar during the first 4–6 days after inoculation to fresh medium, though strain IOW94 proliferated one to two days slower under the same conditions, and tends to aggregate to clumps of bacteria. On days 2 to 3, strains demonstrated solitary cells on a stalk of different lengths (Figures 5, 6). On days 3 to 4, the development of two-cell colonies appeared (Figure 6A). Such colony types were common for IOW73, and are also typical for *Codosiga gracilis* de Saedeleer, 1927 (basionym *Monosiga gracilis* Kent, 1880), but with larger cell dimensions. Strain IOW94 normally produced 2–4 cell colonies, though occasionally largely colonies were formed.

Strain IOW94 was present as sedentary stalked solitary cells and as colonies. It has a flask-shaped cell with a broad and short neck covered with a very delicate, tightly enveloping, theca (see ultrastructure below). The inconspicuous profile of the theca opening is visible in some cells as “whiskers” at the base of the collar (Figure 5A, arrowheads). Length of the body is 3–4.5 μm, width - 2 μm (n = 18). The length of the collar is equal to the body length, the flagellum is approx. 2 times longer than the body and the stalk covers up to 3 body lengths.

Strain IOW73 was present as sedentary stalked solitary cells and as colonies of 2–4 cells (Figure 6A). The most typical colonies were two cells on a rather long stalk (up to 7 μm). The strain has an elongated vase-shaped cell with a narrow and prominent neck, surrounded with a delicate, tightly enveloping, theca (see ultrastructure) with visible whisker. The body length is 2–4 μm, width - 1 μm (n = 22). The length of the collar is equal to the body; the flagellum is 1.5-2 times longer than the body.

The cell shape of both strains is similar to *C. gracilis*, studied by Leadbeater and Morton [28]. A contractile vacuole was not visible for cells cultivated at 22 ‰ but appeared when the salinity was reduced to 8–10 ‰ (Figure 6A, B).

### Ultrastructure

The electron microscopical investigations revealed an in general typical choanoflagellate cell structure for both strains (Figures 5, 6). As in many others colonial choanoflagellates: (1) the cells were covered with a thin sheath, which envelopes the whole body and the base of the collar (Figures 5A, B, 6B); (2) the collar was composed of approximately 30 microvilli in both isolates (not shown); (3) the Golgi apparatus lies under the base of flagellum (Figure 5B); (4) the flagellar apparatus has a long transition zone, a flagellar kinetosome with radiating microtubules, and a non-flagellar centriole, all typical for choanoflagellates (Figure 5B, 6D); (5) a nucleus of vesicular type (Figure 6B) is located in the anterior-middle part of the cell; and (6) other organelles and inclusions are also those common for choanoflagellates. Additionally, food vacuoles with bacteria in different stages of digestion were found in the posterior half of the cell, and a contractile vacuole is located at the cell posterior. This latter structure has the typical appearance of a folded reservoir with coated pits and vesicles around it (Figure 6B). Finally, lipid droplets occur in the cytoplasm of some cells (Figures 5D, G, 6C).

In contrast to these similarities, the internal structure of mitochondria—the shape of the cristae—is cardinally different from all other choanoflagellates investigated to date. The cells in both strains have mitochondria with tubular or sac-like cristae (Figure 1B including left upper insert, 5F, G, 6B insert lower left). In both types the cristae have tubular or saccular shape (Figure 5B, F, G). In the strain IOW94 mitochondria of two types can be seen: with normal matrix and developed cristae (Figure 5B, F), and with light matrix and rare cristae (Figure 5G).

Another peculiarity is the presence of many intracellular, potentially symbiotic bacteria in the cytoplasm of strain IOW94, predominantly in the cell posterior (Figure 5C). These prokaryotes are not limited with membranes, instead lying freely in the cytosol, and seem to belong to Gram-negative bacteria (Figure 5C, D, G) due to the two covering membranes (Figure 5D). They are represented by at least two types: long narrow (nlb) and big flagellated bacteria (bfb). The bfb have a set of rather long flagella which are tubular in cross section (Figure 5D) and tend to associate with lipid globules (Figure 5D, E, G).

### Mode of feeding

Live observations of both strains revealed a typical *Monosiga*-type mode of feeding [29,30]. The feeding pseudopodium arises from the top of the neck outside the collar, grows towards the bacterium on the outer surface of the collar and engulfs the prey producing a food vacuole. These observations were confirmed by cross sections through the collar base (Figure 6B, insert). Additionally, feeding pseudopodia arising from the side of the neck were found for both strains (Figure 6C). This mode of engulfment is typical for *Codosiga* and some other colonial choanoflagellates with a thin sheath around the cell [29,30]. The presence of two feeding modes is easily explained by the combination of solitary and colonial life styles for both strains: solitary cells feed in *Monosiga*-type mode, and colonial cells feed as other colonial choanoflagellates (*Codosiga, Desmarella, Sphaeroeca*).

### Formal taxonomic description

*Codosiga balthica* sp. nov. Wylezich et Karpov (Choanoflagellatea (Kent) Cavalier-Smith, 1998, Craspedida Cavalier-Smith, 1997; Salpingoecidae (Kent) Nitsche et al., 2011).

Diagnosis: Sedentary stalked solitary cells with rare production of colonies of 2–4 cells. Flask-shaped cell with a broad and short neck surrounded by a delicate sheath, visible through electron microscopy. Dimensions: body length - 3–4.5 μm, width - 2 μm, length of the collar equal to the body, flagellum 2–2.5 times longer than the body, stalk: up to 3 body lengths. Tubular or saccular mitochondrial cristae, intracellular flagellated bacteria present in cytosol not limited with membrane. Observed habitat: Gotland Deep (central Baltic Sea, IOW station 271, 57°19′N, 20°10′E) suboxic to anoxic water masses (depth 206 m), brackish (8–25 ‰); Type material: iconotypes: Figure 5D, E; fixed and embedded specimens (hapantotypes) are deposited at the Oberösterreichische Landesmuseum in Linz, Austria (inventory number 2012/121); live strains (paratypes) are held as clonal cultures (strain IOW94) in the laboratory of the Leibniz Institut for Baltic Sea Research in Rostock-Warnemünde; Etymology: *balthica* after the Baltic Sea, where the strain was isolated. Closely related clonal sequences were available from Gotland Deep and Framvaren fjord but not from other habitats, oxic or hypoxic.

*Codosiga minima* sp. nov. Wylezich et Karpov (Choanoflagellatea (Kent) Cavalier-Smith, 1998, Craspedida Cavalier-Smith, 1997; Salpingoecidae (Kent) Nitsche et al., 2011).

Diagnosis: Sedentary stalked solitary cells which rarely produce colonies of 2–4 cells. Elongated vase-shaped cell with a prominent neck, surrounded by a delicate sheath visible through electron microscopy. Dimensions: body length - 2–3 μm, width - 1 μm, length of the collar equal to the body, flagellum 1,5-2 times longer than the body, stalk is up to 7 μm. Profiles of the mitochondrial cristae of oval shape. Observed habitat: Gotland Deep and Landsort Deep (central Baltic Sea, IOW station 284, 58°35′N, 18°14′E) suboxic to anoxic water body (depths see Table 1), facultative anaerobic, brackish (8–16 ‰); Type material: iconotypes: Figure 6B and insertion down left; fixed and embedded specimens (hapantotypes) are deposited at the Oberösterreichische Landesmuseum in Linz, Austria (inventory number 2012/120); live strains (paratypes) are held as clonal cultures (strains IOW73-75) in the laboratory of the Leibniz Institut for Baltic Sea Research in Rostock-Warnemünde; Etymology: *minima*, due to the small cell size.

**Table 1:** Isolated strains, with the corresponding isolation depths and physico-chemical data (Gotland (G) and Landsort Deeps (L), central Baltic Sea) and GenBank accession numbers for partial gene sequences generated in this study

**Species**	***Codosiga balthica***	***Codosiga minima***		
Detected via	Clone library (**G**^1^) DGGE (**G**^2^, **L**^3^) Isolation (**G**^4^)	Isolation (**G**, **L**^4^)		
Strain	IOW94	IOW73	IOW74	IOW75
Station	271 (**G**)	271 (**G**)	271 (**G**)	284 (**L**)
Depth [m]	206	150	208	260
O_2_ [μM]	0.85	1.57	0.48	4.23
H_2_S [μM]	0.13	0.25	1.77	n.det.
18S rRNA	JQ034424	JQ034422	n.sub.	n.sub.
28S rRNA	JQ034425	JQ034423	n.det.	n.det.

^(1^Stock *et al*. 2009 [20]; ^2^Weber 2008 [37]; ^3^Anderson *et al*. [38] (in revision); ^4^this study; n.det., not detected; n.sub., not submitted to GenBank).

Remarks. The species described here could easily be separated from *C. gracilis* based on their size (2–4.5 μm length for IOW73 and IOW94 vs. 4–8 μm for *C. gracilis*), the shorter flagellum (max. 8 μm vs. 8–20 μm for *C. gracilis*), the flagellar root microtubules (organised in one row vs. 2–3 rows for *C. gracilis*[28,30,31]) and the shape of mitochondrial cristae. *C. balthica* differs from *C. minima* by possessing intracellular bacteria and based on 18S and partial 28S rRNA gene sequences. No 18S rRNA sequence of *Codosiga* cultures exists (as discussed in [6]), but the clustering of the 28S rRNA tree supports the separation of both our strains from their nearest neighbour, *C. gracilis* (Figure 4). Both species descriptions are deposited in ZooBank under urn:lsid:zoobank.org:act:8EA52C91-58CE-4FF9-9007-AC9DED267DD6 (*C. minima*) and urn:lsid:zoobank.org:act:DF26A642-BD7A-4819-BE8C-40B01A1E7971 (*C. balthica*).

## Discussion

Putative anaerobic choanoflagellate species have been occasionally detected using microscopical methods [32,33]. For example, *Diaphanoeca* sp. and *Acanthocorbis* sp. were found in fixed samples from suboxic to anoxic/sulfidic waters of the Mariager Fjord [9] but did not grow in anaerobic incubations. In contrast, *Codosiga* species had not been described to date for hypoxic environments.

As shown here, aloricate choanoflagellates (including choanoflagellate cells that show no lorica under epifluorescence microscope) in general are numerically important members of the Baltic redoxcline protistan community with a peak at the suboxic zone above the chemocline. Their relative abundance was higher in Gotland Deep (up to 20 to 30% of total HNF cell-counts) than in Landsort Deep (up to 5%). The Gotland Deep is characterized by periodical small-scale mixing events [34,35] and frequent lateral intrusions of oxygenated water [20,36], which lead to a less stable redoxcline than in Landsort Deep. Nevertheless, both deeps are rather similar concerning salinity, oxygen and sulfide content and should principally be colonized by both species if they are tolerant to anoxic and sulfidic conditions and it requires more samplings to reveal consistent differences in the spatial and temporal distribution of the two species.

The single cell isolations, conducted in 2005, gave us the opportunity to isolate and describe strains from these abundant choanoflagellates. On the same cruise, redoxcline samples from Gotland Deep were collected for RNA-based clone library investigations of oxic-anoxic transition zone and sulfidic water depths [20] which resulted in several 18S rRNA clonal sequences highly similar to our *C. balthica* isolate (see framed clade in Figure 3). RNA-based clone libraries can be influenced by different numbers of ribosomal RNA molecules depending on cell size, trophic state or rather metabolic activity. Because of the small cell size of *Codosiga* spp. we would expect that its contribution in clone libraries of the total protistan community is only minor. However, the high amount of clonal sequences closely related to *C. balthica* found by Stock *et al*. [20] (11% and 4% in the library of the oxic-anoxic transition zone and the sulfidic zone, respectively) indicates in our opinion a high abundance of the corresponding cells at the sampling site. The 18S rRNA sequence of *C. balthica* also was reported via DGGE fingerprint techniques from the same habitat in 2007. The relevant DGGE band was detected only in water depths below the chemocline, representing anoxic/sulfidic water layers until concentrations of 11 μM hydrogen sulfide [37]. These data correspond to the vertical distribution of *Codosiga* spp. at the sampling time (Figure 1), where they were mainly found in anoxic depths. Additionally, an identical sequence was detected from a DGGE fingerprint from Landsort Deep permanent redoxcline collected at the oxic/anoxic interface in 2011 38. Overall, our results indicate that at least *C. balthica* is a permanent and prominent member of the protistan community of Gotland and Landsort Deep redoxclines.

In contrast to this taxon*, C. minima* was isolated for cultivation from three different redoxcline samples during a cruise in 2005. Two of these cultures were obtained from Gotland Deep (strain IOW73, 150 m; strain IOW74, 208 m) while the third isolate came from Landsort Deep (strain IOW75, 260 m; identity seen at an 18S rRNA sequence level, see Table 1). In contrast to *C. balthica*, no closely related environmental sequence for *C. minima* was found in GenBank, which is typical for several isolated and cultivated protistan taxa with presumably only minor ecological relevance [39,40].

The general ultrastructure of both species described here is similar to that of other investigated “naked” craspedids [41-43]. However, the singular adaptation of their mitochondria, and, in the case of *C. balthica*, the acquisition of intracellular bacteria, are very likely strategies gained both species to deal with oxygen depletion.

The cells of *C. minima* have mitochondria with tubular but developed cristae, while *C. balthica* has mitochondria of two types: m1 and m2 (see Figure 5). Both types of mitochondria have predominantly cristae with a tubular shape, but the type m2 shows a reduced number of cristae and an electron translucent matrix. Tubular cristae have never been found before in choanoflagellates, even in specially designed experiments to change the shape of mitochondrial cristae with steroids, conducted unsuccessfully on a *M. ovata* culture [44]. Mitochondria with reduced number of cristae were recently classified as anaerobically functioning mitochondria of the class 2 [45]. Such mitochondria have a reduced enzyme inventory with regard to oxidative phosphorylation and are able to use other electron acceptors than oxygen (e.g. fumarate or nitrate). The routine growth of our strains under normoxic circumstances in the laboratory shows that the mitochondria of both species can use oxygen without any difficulty. It is not clear at the moment whether the two types/classes of mitochondria in *C. balthica* coexist permanently or if some of the mitochondria transformed into aerobically functioning ones (class 1 according to Müller *et al*. [45]) during the cultivation under oxic condition. Higher numerical reduction of cristae (oxygen consuming components) in *C. balthica* mitochondria class 2 and the abundance of this taxon in oxygen depleted waters support the possibility to use other electron acceptors in response to decreasing oxygen levels in the environment.

Prokaryotic endosymbionts are common in protists, particularly in ciliates and dinoflagellates [46,47], but had never been observed previously for choanoflagellates [41-43]. Anaerobic ciliates often harbour methanogenic archaeans in close connection to their hydrogenosomes, and Eubacteria without connections to the hydrogenosomes [48,49]. *C. balthica* clearly does not possess hydrogenosomes and its endobionts are of bacterial nature as recognizable by the second enveloping membrane instead of a cell wall like archaeans (Figure 5D). Interestingly, the intracellular prokaryotes were not lost during nearly seven years of cultivation under oxic conditions, indicating that this is likely an obligate symbiosis for the choanoflagellate. Similar to observations in anaerobic ciliates, the endobionts likely support the choanoflagellate host (*C. balthica*) during anaerobic metabolism and thus allowed them to colonize oxygen depleted zones that supply high food availability. However, at this time we can not further specify the identity and role of these intracellular prokaryotes.

As noted in the introduction, environmental choanoflagellate sequences are typical constituents of pelagic redoxcline protist communities and have been frequently detected in hypoxic waters via clone libraries [18-20,50,51]. One environment in particular is worthy of mention: although the Cariaco Basin is globally the most comprehensively sampled redoxcline environment (nearly 7,000 entries in GenBank of partial clonal 18S rRNA gene sequences for this habitat; e.g., [50,52,53]), no sequences belonging to *C. balthica* or *C. minima* have been found there. This could be deeply rooted in methodological limitations (e.g. different primers used for RNA or DNA templates). Alternatively, the higher salinity of the Cariaco Basin, or other physico-chemical or hydrological parameters, could exclude the two Baltic *Codosiga* species from this environment with fully saline conditions. However, these species seem to be relatively insensitive to salinity variations and are highly tolerant to the presence of oxygen and sulfide. They were able to grow in culture at 8 ‰ (this study) and one sequence related to strain *C. balthica* comes from deeper hypoxic water layers of the Framvaren Fjord at about 25 ‰, [18]. Thus, the possibility that these species represent endemic taxa of the Baltic Sea region should be taken into consideration and will be tested in further studies.

## Conclusions

Both isolated species described here, *C. minima* and *C. balthica*, were found within suboxic to anoxic water layers, in the latter case using different approaches and in several years. The species are of interest due to their habitat, from which no choanoflagellate cultures could be obtained yet, their unusual mitochondrial cristae and presence of intracellular prokaryotes in one species. Our isolation effort is important in view of the complexity of isolation and cultivation of choanoflagellates species [5] and of protists that can survive in hypoxic environments in general. The novel *C. balthica* is ecologically relevant component of the protist community at the sampling sites tested. With its interior (derived mitochondria, prokaryotes), at least *C. balthica* is potentially able to outcompete less adaptable heterotrophic nanoflagellates and to become abundant in hypoxic parts of the Baltic Sea. Preliminary investigations have shown that *C. balthica* is able to grow successfully under suboxic conditions in the laboratory, but not *C. minima* (M. Marcuse, C. Wylezich & K. Jürgens, unpublished results). Our next challenges would be (1) to identify and characterize the functional role of the intracellular prokaryotes of *C. balthica*, and (2) to determine the quantitative contribution of both species to the Baltic protistan community via fluorescently labelled specific probes. Moreover, both cultivated species are ideal model organisms for future studies on temporary anaerobic metabolism using derived mitochondria.

## Methods

### Sampling, isolation/cultivation and counting of choanoflagellates

Strains of the newly described *Codosiga* spp. were obtained from untreated plankton samples taken in the central Baltic Sea at the Gotland (IOW-station 271; 57° 19.2′ N; 20° 03′ E) and the Landsort Deep (IOW-station 284; 58° 35.0′ N; 18° 14.0′ E) in May 2005 during an expedition with the RV *Alkor*. Clonal cultures were obtained from a single cell shortly after sampling, which was isolated using a micromanipulator fitted with glass micropipette [54]. The cultures were deposited as part of the IOW culture collection, and were routinely kept in sterile 50-ml tissue culture flasks (Sarstedt, Nümbrecht, Germany) in F2 medium [55] (salinity 8–12 ‰) on a mixture of bacteria grown on a wheat grain. Altogether four choanoflagellate cultures could be established (Table 1).

Samples for cell-counts of HNF were obtained on board the RV *Poseidon* in August 2008 (Gotland Deep) and the RV *Maria S. Merian* in September 2009 (Gotland and Landsort Deep). Water from different depths (GD 2008: 114–137 m, GD 2009: 90–140 m, LD 2009: 70–120 m) was collected in 10 l free-flow bottles attached to a conductivity, temperature and depth rosette (CTD) with a coupled oxygen sensor. In all cases, oxygen and hydrogen sulfide were measured immediately on board according to standard methods [56]. In order to avoid potential oxygen contamination during emptying of the free-flow bottles, for experimental purposes only the bottom 5 l of water from 10 l free-flow bottles was employed.

### Molecular biological investigations

DNA was extracted from cells harvested from 20–30 ml of dense cultures (8000 g, 20 min, 4°C) using a CTAB extraction as described previously [57]. The 18S rRNA gene was amplified by polymerase chain reaction (PCR) using eukaryotic specific primers 18SFor-n2 (5′- GAT CCT GCC AGT AGT CAT AYG C - 3′) and 18SRev-Ch (5′- TCC TTC TGC AGG TTC ACC TAC GG - 3′). The mixture containing 0.1 mM of each primer, 200 mM dNTPs, 10 mM Tris pH 8.3, 1.5 mM MgCl2, 50 mM KCl, and 1 unit of *Taq* DNA polymerase (Fermentas) was heated to 95°C for 2 min, and the 18S rRNA gene was amplified in 35 cycles of 95°C for 30 s, 52°C for 45 s, and 72°C for 2 min, followed by 10 min at 72°C. PCR products were purified with the Nucleospin II Kit (Machery Nagel). Sequencing was carried out by a company (Qiagen) with the primers used for PCR and four different internal sequencing primers (590F: 5′- CGG TAA TTC CAG CTC CAA TAG C - 3′, 600R: 5′- GCT ATT GGA GCT GGA ATT ACC G - 3′, 1280F: 5′- TGC ATG GCC GTT CTT AGT TGG TG - 3′, 1300R: 5′- CAC CAA CTA AGA ACG GCC ATG C - 3′). The 28S rRNA was amplified as described above with extended elongation time (3 min) annealing of 48°C using primers fw1 (5′- AGC GGA GGA AAA GAA ACT A - 3′) and 20R (5′- GAG AGT CAT AGT TAC TCC C - 3′, kindly provided by C. Berney). The purified PCR products were partially sequenced by use of primers 1274 (5′- GAC CCG TCT TGA AAC ACG GA - 3′), D5-Rev2 (5′- GGC AGG TGA GTT GTT ACA - 3′, all given in [57]), and the newly designed primer D2D3-Rev (5′ - GAC TCC TTG GTC CGT GTT TC - 3′).

Obtained sequences were checked and corrected using Bioedit [58]. Genetic distances were calculated with Mega [59]. Sequences were aligned together with other sequences retrieved from GenBank using Clustal_X program [60]. Afterwards, the alignments were edited manually. Two data sets of the sequence alignments were created for the 18S and 28S rRNA gene sequences. The 18S rRNA data set contains 1,623 aligned nucleotide positions, and the 28S rRNA alignmet excluding the high divergent D2 region was 1,497 positions in length. We used MrBayes [61] and PhyML 3.0 (http://www.atgc-montpellier.fr/phyml/[62]) for the phylogenetic analyses. The analyses were done using the GTR model of substitution [63] and gamma-shaped distribution of rates of substitution among sites with eight rate categories. The Bayesian analysis was performed for 1,000,000 generations and sampled every 100 generations for four simultaneous MCMC chains (born-in = 2,500). For the maximum likelihood analysis all model parameters were estimated from the data set. To estimate branch support, we performed 500 bootstrap replicates for maximum likelihood analyses. Phylogenetic reconstruction based on the partial 28S rRNA gene we chose choanoflagellate sequences from GenBank that cover the complete length of sequence fragments generated in this study.

### Microscopical investigations

For light microscopy observations of living cells a DM 2500 microscope (Leica) was used. For electron microscopy, the cultures were adapted to a salinity of 8 ‰ to simplify the fixation protocol. The cell-pellet was fixed, on ice in the dark for 30 min, with a cocktail containing 2% glutaraldehyde and 1% osmium tetroxide in F2 medium, buffered with 0.05 M cacodilate to pH 7.2. After dehydration in an alcohol series the pellet was embedded in Epon/Araldite resin, sectioned with a glass knife, and stained with uranyl acetate and lead citrate. The sections were observed at 80 Kv, under an EM Margani FI 268 electron microscope equipped with digital camera (Olympus Megaview III).

For flagellate identification in 2005, a combination of live observations and scanning electron microscopy was employed. For live samples, sea water was concentrated by reverse filtration (0.2 μm membrane filter; Millipore GmbH, Schwalbach, Germany) in a hermetic box with a nitrogen atmosphere at 4°C. Concentrated samples were then placed inside a 1 ml transparent glass chamber, hermetically sealed with a cover slip, and observed, directly on board, using phase contrast at 360X and 630X under an Axiovert 40 CFL inverted microscope (Carl Zeiss MicroImagimg GmbH). Additionally, individual flagellate cells were isolated by means of a specially constructed micropipette [54], and cultured in 96-well plates or petri-dishes, with sterile autoclaved Baltic Sea water as medium and *Pseudomonas putida* MM-1 as food source. Dried whole mount preparations of these flagellates were later examined with a JEM-1011 transmission electron microscope (JEOL Ltd.; Tokyo, Japan) as previously described [64].

For HNF cell counts in 2008 and 2009, 100 ml samples were fixed with a final concentration of 1% particle free formaldehyde in brown glass bottles, at 4°C, between 2 and 24 h. Subsamples were filtered onto black polycarbonate filters (0.8 μm pore-size; 25 mm diameter; Whatman GmbH, Dassel, Germany), which were stored at −20°C or −80°C. Filters were later stained with DAPI at a concentration of 0.01 mg ml^−1^, mounted, and observed under a Zeiss Axioskop 2 mot plus epifluorescence microscope (Carl Zeiss MicroImagimg GmbH, Gottingen, Germany). A minimum of 100 cells per filter were counted at 630X using filter set 02 (Carl Zeiss MicroImagimg GmbH). Aloricate choanoflagellates were clearly distinguishable and therefore counted as a separate group.

## Abbreviations

18S rRNA: Small subunit of ribosomal RNA; 28S rRNA: Large subunit of ribosomal RNA; ag: Golgi apparatus; bfb: Endobiotic big flagellated bacteria; CTAB: Cetyl trimethyl ammonium bromide; cv: Contractile vacuole; DGGE: Denaturing gradient gel electrophoresis; EM: Electron microscopy; f: Eukaryotic flagellum; fl: Prokaryotic flagella; fv: Food vacuole; GD: Gotland Deep; GTR: General time reversible; HNF: Heterotrophic nanoflagellates; kn: Flagellar kinetosome; l: Lipid droplet; LD: Landsort Deep; LM: Light microscopy; m: Mitochondrion; m1: Mitochondrium type 1; m2: Mitochondrium type 2; MCMC: Markov Chain Monte Carlo; mt: Microtubules of flagellar root system; mv: Microvilli of the collar; n: Nucleus; nlb: Narrow long endobiotic bacteria; nu: Nucleolus; ps: Food pseudopodium; st: Stalk.

## Competing interests

The authors declare that they have no competing interests.

## Authors’ contributions

CW generated the 18S and 28S rRNA gene sequences, carried out the phylogenetic analyses and wrote the first draft of the paper; SK generated the LM and TEM data and interpreted these data and contributed to writing the manuscript; APM collected and isolated the specimens for cultivation, and analysed its vertical distribution in 2005; RA did sampling, counting and analyzing of HNF and choanoflagellates in 2008 and 2009 and contributed to writing the manuscript; KJ funded the flagellate collection, organized the cruises and contributed analytic tools; all authors have read, edited and approved the final manuscript.
